# Optimal Deep Learning Architecture for Automated Segmentation of Cysts in OCT Images Using X-Let Transforms

**DOI:** 10.3390/diagnostics13121994

**Published:** 2023-06-07

**Authors:** Reza Darooei, Milad Nazari, Rahele Kafieh, Hossein Rabbani

**Affiliations:** 1 Medical Image and Signal Processing Research Center, School of Advanced Technologies in Medicine, Isfahan University of Medical Sciences, Isfahan 8174673461, Iran; reza.darooei@gmail.com (R.D.); rkafieh@gmail.com (R.K.); 2 Department of Bioelectrics and Biomedical Engineering, School of Advanced Technologies in Medicine, Isfahan University of Medical Sciences, Isfahan 8174673461, Iran; 3 Department of Molecular Biology and Genetics, Aarhus University, 8200 Aarhus, Denmark; mina@dandrite.au.dk; 4 The Danish Research Institute of Translational Neuroscience (DANDRITE), Aarhus University, 8200 Aarhus, Denmark; 5 Department of Engineering, Durham University, South Road, Durham DH1 3RW, UK

**Keywords:** OCT, semantic segmentation, X-let, cyst

## Abstract

The retina is a thin, light-sensitive membrane with a multilayered structure found in the back of the eyeball. There are many types of retinal disorders. The two most prevalent retinal illnesses are Age-Related Macular Degeneration (AMD) and Diabetic Macular Edema (DME). Optical Coherence Tomography (OCT) is a vital retinal imaging technology. X-lets (such as curvelet, DTCWT, contourlet, etc.) have several benefits in image processing and analysis. They can capture both local and non-local features of an image simultaneously. The aim of this paper is to propose an optimal deep learning architecture based on sparse basis functions for the automated segmentation of cystic areas in OCT images. Different X-let transforms were used to produce different network inputs, including curvelet, Dual-Tree Complex Wavelet Transform (DTCWT), circlet, and contourlet. Additionally, three different combinations of these transforms are suggested to achieve more accurate segmentation results. Various metrics, including Dice coefficient, sensitivity, false positive ratio, Jaccard index, and qualitative results, were evaluated to find the optimal networks and combinations of the X-let’s sub-bands. The proposed network was tested on both original and noisy datasets. The results show the following facts: (1) contourlet achieves the optimal results between different combinations; (2) the five-channel decomposition using high-pass sub-bands of contourlet transform achieves the best performance; and (3) the five-channel decomposition using high-pass sub-bands formations out-performs the state-of-the-art methods, especially in the noisy dataset. The proposed method has the potential to improve the accuracy and speed of the segmentation process in clinical settings, facilitating the diagnosis and treatment of retinal diseases.

## 1. Introduction

OCT is a non-invasive and powerful imaging technique in ophthalmology that produces micrometer-resolution 3D images of structural and molecular of biological tissues within the human retina [1,2]. It is a very effective diagnostic technique that offers information on the structure and function of the eye, allowing ophthalmologists to diagnose and monitor a variety of eye illnesses [3]. OCT is often employed in clinical settings to track the development of retinal diseases and emerged as the gold standard for diagnosing the most common macular disorders, including Diabetic Macular Edema (DME) and Age-Related Macular Degeneration (AMD). One of the main causes of blindness and visual impairment is fluid leaking from damaged retinal blood vessels, which results in AMD and DME. Macular Edema (ME) occurs when the blood vessels that leak into the center of the retina swell as a result of a buildup of fluid. Ophthalmologists may take necessary efforts to avoid vision loss and offer better results for their patients if these alterations are detected in their early stage of development [4,5].

A cyst is a tiny, fluid-filled object that appears on ophthalmic OCT images of abnormal eyes [6]. Cysts may be discovered in numerous sections of the eye, including the retina, choroid, and anterior chamber. They can indicate various eye disorders, including Retinal Detachment, Macular Edema, and AMD [7]. The aperture enlarges over time and affects the patient’s eyesight. Three subcategories are presented based on the location of the cyst that is characterized by the development of abnormal blood vessels in the choroidal vasculature: pigment epithelial detachment, subretinal fluid, and intra-retinal fluid. Cysts in the eye may have a variety of effects on vision and eye health, depending on their location and size, as well as the underlying eye disease. Cysts in the retina, for example, may cause vision loss if the retina detaches, while cysts in the choroid can indicate macular edema or AMD [8]. Early detection and precise monitoring are the key factors to avoid blindness in these patients. Ophthalmologists are interested in accurately segmenting the Macular Edema-affected areas. Furthermore, the size, shape, and locations of cysts are very different from case to case [6]. Manual cyst segmentation in OCT images can be a time-consuming and tedious process, and it also requires expertise to accurately identify and segment the cysts. This fact is why automated methods using deep learning and other image-processing techniques were developed in this task to assist researchers and clinicians in the field of ophthalmology [9].

In computer vision systems, semantic segmentation is a complex process to complete. Many strategies were previously created to address this issue [10]. Semantic segmentation is the ability to segment an unknown image into several components and objects and assign a class label to each pixel. In recent years, a number of automated segmentation techniques were suggested. For problems involving the segmentation of the cysts, traditional machine-learning techniques were applied extensively in research that began in the 2000s [10]. Traditional methods for OCT cyst segmentation typically involve pre-processing the image to enhance the contrast and applying thresholding and morphological operations to segment the cysts. These methods are relatively simple and computationally efficient; however, they often struggle with the complex shape and texture of the cysts, as well as the presence of noise and artifacts in the OCT images [11]. Traditional cyst segmentation methods, such as Thresholding [11], Graph-Based Methods [12,13], and Active Contour Methods [14], can be useful; however, deep learning methods were shown to improve segmentation performance in various medical imaging applications, including OCT cyst segmentation. Deep learning models can learn and extract features automatically from the input data, reducing the need for manual feature engineering, and can achieve high accuracy in segmentation tasks [11,15]. Our main goal, in this paper, is to show how using appropriate sparse basis functions can improve the performance of Unet as a basic cyst segmentation unit due to the consistency of chosen basis functions to the properties of cystic B-scans.

## 2. Related Works

In this section, the required background about the deep learning methods and directional X-lets is presented. Deep learning gained significant attention in recent years as it demonstrated remarkable performance in image segmentation [16]. Moreover, directional X-lets are a family of transforms that capture directional information in images [17]. In this section, we provide an overview of these methods and their applications in the field of ophthalmology, particularly for the cyst segmentation of retinal OCT images.

### 2.1. Deep Learning Review

Deep learning recently made major breakthroughs in the area of medical imaging due to rising computer power and the amount of available data [18,19]. Convolutional Neural Network (CNN) structures, in particular, can capture the non-linear mapping between inputs and outputs, as well as automatically learn local area features and high-level abstract features via multi-layer network structures, which are typically superior to a manual extraction and pre-defined feature sets [19]. Various deep learning techniques were used to segment retinal layers and retinal lesions. For example, some of the most advanced deep learning architectures for image segmentation are FCNN [20], R2 Unet [21], Seg-net [22], and Deeplabv3+ [23]. Trans-Unet was developed to identify fluid in our earlier work [24]. Trans-Unet is similar to Unet [20,25]. It comprises an expanded path and a contracting path. It uses a hybrid CNN-Transformer architecture to make use of both the global context stored by transformers and the finely detailed high-resolution spatial information from CNN features.

Deep learning is used in ophthalmology to make use of the large datasets of the fundus, while for OCT images that are frequently obtained in clinics [16], particularly for computer-aided diagnosis, deep learning-based automated detection of glaucoma is often performed on wide-field OCT scans [26]. Deep learning was also utilized in fundus and OCT images [26] for the segmentation and classification of retinal vasculature and retinal layers [27]. Fully Convolutional Neural Networks (FCN) show outstanding performance in image segmentation challenges [28]. There were a few past attempts to use FCNs for retinal fluid segmentation [29].

Fully Convolutional Neural Networks, Adversarial Networks, and Unets are some of the most popular methods used for OCT cyst segmentation tasks. Unet has several advantages over fully convolutional methods that make it a better choice for certain tasks, especially in medical imaging. Although Unet is a FCN method, some improvements could help Unet performs more effectively [30]. One advantage of Unet is that it includes both a contracting path and an expanding path, which allows for the precise localization of features. The contracting path is a sequence of convolutional and max pooling layers that reduce the spatial dimensions of the input. In contrast, the expanding path is a sequence of up-convolutional layers that increase the spatial dimensions. This structure enables Unet to effectively capture both high- and low-level features, allowing for a more detailed segmentation [31,32]. Moreover, adversarial approaches are employed in semantic segmentation, especially in medical imaging in which is the size of the artifact is too small others; however, Unet-based methods are thought to outperform adversarial approaches owing to their ability to properly localize objects while preserving fine-grained information [10,33]. Overall, Unet’s sophisticated architecture and its ability to effectively preserve and utilize both high- and low-level features make it a better choice than FCN and adversarial methods for certain tasks; thus, we will use Trans-Unet as the base network for the sub-bands analysis.

The succeeding pooling layers minimize the spatial information of the feature in order to learn more abstract feature representations [34]. However, dense prediction problems need extensive spatial information. Due to the limitations of standard pooling techniques, they are (1) prone to disregard essential texture information in the image and (2) inadequately resilient to noise. The sub-bands employ a spectral domain transformation approach to address these difficulties.

### 2.2. X-Lets Review

X-lets refer to a broad category of signal-processing techniques that use mathematical functions or transforms to analyze and manipulate signals [17]. X-lets such as wavelets and circlets, contourlets, and Dual-Tree Complex Wavelet Transform (DTCWT) have various applications in signal processing and image analysis, including denoising, edge detection, and compression. X-lets are particularly useful for signal and image denoising because they can represent both the high- and low-frequency components of a signal with a variable window size, which allows for more accurate noise reduction while preserving important signal features. X-lets could be useful for image segmentation because of the specific time–frequency properties of different sub-bands. X-let sub-bands have low computational complexity and better preserved edges in images, making them a promising tool for several applications [17].

In deep learning frameworks, X-let transforms can be applied in two ways: (1) as part of the network architecture (e.g., using wavelet layers instead of pooling layers); or (2) using sparse X-let representations of the image as inputs in the network. The X-let transform was used to overcome problems with pooling in deep learning. Conventional down-sampling methods, such as max-pooling and average pooling, usually ignore the Nyquist sampling theorem; however, anti-aliased CNNs can improve segmentation accuracy by incorporating the wavelet transform [35]. Moreover, as shown in similar studies, other X-lets could easily use this structure. Hongya Lu et al. segmented human thyroid applications using DTCWT-based CNN. They attempted to apply DTCWT in CNN layers rather than the max-pooling layer [35]. Similarly, Qiufu Li et al. developed a modified Unet model with a Wavenet wavelet layer. André Souza’s approach improved performance, while Brito et al. coupled max-pooling with wavelet pooling. This group proposed a novel multi-pooling approach that combined wavelet and classic pooling. Alijamaat et al. attempted to combine wavelet pooling with Unet to improve semantic segmentation performance in order to extract distinct directions in brain MRI images [30]. Guiyi Yang et al. [36] used wavelet transforms in the Attention Unet for concrete crack segmentation. Several researchers tried to integrate the wavelet transform into deep learning models, such as the Unet and Attention Unet models. Some studies applied the wavelet transform in the network structure, while others have used sub-bands as the input image. For example, one team used contourlet sub-bands as inputs in their marine raft segmentation network based on Attention Unet [37]. Another team utilized 3D discrete wavelet transform as the input image for polarimetric SAR images [38]. To the best of our knowledge, no prior research examined the different X-let transform configurations and function of each sub-band in OCT cyst segmentation. Table 1 displays of different sparse-based deep learning methods are discussed in the related works.

This paper focuses on using X-let sub-band combinations in a deep-learning framework to segment OCT fluid. X-lets are effective time–frequency analysis tools that decompose an image signal at different time scales using a family of basic functions. They provide improved edge preservation and low computational complexity compared to other wavelet transforms.

We aim to compare the effects of different sub-band combinations in terms of performance metrics, such as the Jaccard index and Dice scores. They examine the impact of different sub-band combination transforms and their new formation types on the deep learning framework. To the best of our knowledge, this is the first comprehensive comparative study on the application of different X-lets sub-bands in OCT fluid segmentation. The use of sub-band combinations in deep learning methods for OCT fluid segmentation has the potential to significantly improve the accuracy of the segmentation results, especially in normal and noisy conditions. By conducting a comprehensive comparison study on the application of different X-let sub-bands, the authors aim to provide a better understanding of the optimal sub-band combination required for OCT fluid segmentation.

In this paper, we attempted to expand on the state-of-the-art approach in a variety of ways:

1. We used the X-lets input framework to enhance the accuracy of semantic segmentation in OCT images and evaluated the performance of various X-let combinations;
2. We proposed a novel optimal X-let transform by concatenating different sub-bands and two-channel sub-bands, which resulted in improved segmentation performance;
3. Our study highlighted the importance of choosing the right architecture and combination of sub-bands for OCT cyst segmentation and provided insights into the strengths and weaknesses of different methods;
4. We compared different methods, architectures, and transforms to choose the best combination and transform required for the OCT semantic segmentation application.

## 3. Materials and Methods

### 3.1. Dataset

The dataset is introduced in this section to evaluate and compare the performance of different X-lets and their combinations (of basis functions) for OCT cyst segmentation. The preparation of the dataset involved acquiring and pre-processing images of OCT B-scans of cysts. Once the images were pre-processed and labeled, the dataset could be split into training and testing subsets to facilitate model training and evaluation. It was crucial to ensure that the dataset was diverse and representative of the various types of OCT images that may be encountered in real-world scenarios. This approach could help increase the robustness and generalizability of the deep learning models trained on this dataset.

Two distinct datasets were used to investigate the impact of various X-let sub-band combinations. The initial dataset included 194 B-scans created via the Heidelberg OCT equipment (fluid and normal). Our team previously obtained this dataset [41]. We needed to enlarge our dataset to boost our performance. Our dataset was integrated with the Retinal OCT Fluid Challenge (OPTIMA) [42]. This collection comprises 356 OCT B-cans images at various resolutions, including normal and fluid images. All images and ground truth masks from the various datasets were scaled to 512 × 512 resolutions. The data may be found at https://github.com/rezadarooei/OCT fluid dataset (accessed on 1 December 2022).

To enhance our dataset and test the agreement and repeatability of the proposed approach network, we employed data augmentation methods, such as rotation, shift, and crop. The datasets were separated into training and test sets at random. The dataset contains 502 different fluid images; using data augmentation, we sourced 5500 standard images. In this process, we applied different random combinations of data augmentation processes. We also used the structural similarity index measure (SSIM) to calculate the similarity between the augmented images, and eliminated the highly similar (redundant) images; after that, we performed a visual inspection to check the dataset.

The resistance against noise is an important aspect of evaluating any segmentation approach. We used various noise levels, since many OCT imaging devices suffered from the speckle noise. In some devices, such as Heidelberg, the images were displayed in the log domain, and the final observed noise was modeled via additive white Gaussian noise. Different amounts of white Gaussian noise were applied to the database images, resulting in a new database with 70% of the images chosen to be noisy and 30% remaining as the original version. This experiment aimed to look at the effect of noise in sub-bands. Furthermore, this dataset was randomly divided into 80% and 20% for training and testing. 

The Signal-to-Noise Ratio (SNR) is a measure of the test of the quality of a signal, which is defined as the share of the signal power relative to the noise power. It is commonly used in image processing and analysis to evaluate the quality of an image or a signal by this metric. The SNR was calculated as the ratio of the power (variance) of the signal to the power (variance) of the noise in the image, as follows:

(1) $$\begin{array}{c} SNR=10{log}_{10}\left(\frac{{\left|\left|A\right|\right|}_{F}^{2}}{{\left|\left|A-B\right|\right|}_{F}^{2}}\right),\\ Frobenius\;norm:\;{\left|\left|A\right|\right|}_{F}^{2}=\Sigma_{i=1}^{N}\Sigma_{j=1}^{M}a_{ij}^{2}. \end{array}$$

**Figure 1:**
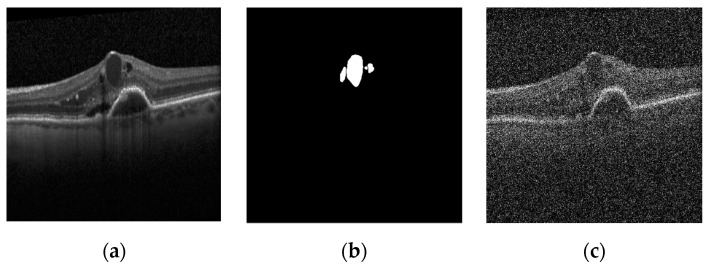
Different dataset images [24]: (**a**) original image; (**b**) true mask; (**c**) noisy image (σ=80).

For this dataset ($\sigma_{n}=80$), the SNR was equal to 8.11 dB. Figure 1 shows a sample image of the dataset, the true segmentation of the cyst on it, and the noise-added image.

### 3.2. X-Let Combination

X-lets (extended wavelets) are a series of mathematical functions that expand regular wavelets, and were discovered to have superior signal and image representation capabilities. We investigated the usage of X-lets as a feature extraction method for automated cyst segmentation in OCT images in this paper. We aimed to increase the performance of deep learning techniques in both normal and noisy situations using the unique qualities of X-lets. The final aim is to identify the optimum X-let foundation for OCT cyst semantic segmentation and compare its performance to other current approaches [17].

Contourlet, dual-tree complex wavelet, curvelet, and circlet are all transform-based image analysis methods that are often used for image processing and segmentation tasks. Each of these methods has unique strengths and weaknesses when it comes to semantic segmentation.

- Contourlet transform: A mathematical method called the contourlet transform is used to find 2D geometries in images. It works by employing the Laplacian Pyramid to divide an image into a number of sub-bands, after which the image is subjected to a number of directional filter banks. The contourlet transform is a multi-scale, multi-directional transform that is specifically designed to handle the edges and contours in an image. This specialization makes it useful for segmenting objects with complex shapes and boundaries. However, the contourlet transform is computationally expensive and may not be suitable for real-time applications [43].
- Dual-tree complex wavelet transforms: The discrete wavelet transform (DWT) breaks down a signal into several sub-bands, with each sub-band representing the signal in a special time–frequency duration. The dual-tree complex wavelet transform is a variation in the standard wavelet transform that provides improved directional selectivity and phase information. DTCWT sub-bands make the transform useful for segmenting objects with textures and fine details. However, the dual-tree complex wavelet transform may not be as effective as the contourlet transform for handling complex corners [44].
- Curvelet transform: The Discrete Curvelet Transform (DCT) is a mathematical tool designed for processing and analyzing digital images. It is based on the concept of curved wavelets, and aims to represent complex structures, such as contours and edges, more efficiently than other popular wavelet transforms. The curvelet transform is a multi-scale, multi-directional transform that is designed to capture the curved and smooth features in an image. This specialization makes it useful for segmenting objects with smooth shapes and boundaries. However, the curvelet transform may not be as effective as the contourlet transform for handling sharp edges and corners [45].
- Circlet transform: The circlet transform is a mathematical signal and image processing tool. It is based on the decomposition of a signal into its component elements, using circular functions as basis functions. When compared to standard linear transforms, such as the Fourier Transform, the transform strives to offer a better depiction of shapes and edges in images. The circlet transform is a recently proposed method that combines the advantages of the curvelet and contourlet transforms. It is designed to handle both sharp edges and smooth contours in an image, making it suitable for a wide range of segmentation tasks. However, the circlet transform is relatively new and is yet to be extensively tested in real-world applications [45].

The choice of transform method for semantic segmentation depends on the specific characteristics of the images being analyzed and the requirements of the application. The choice of sub-bands depends on the application and the type of features that are important for the task. To compare the performance of these sub-bands for OCT cyst segmentation, we evaluated their individual and combined contributions. The contourlet transform is effective for analysis of complex contours, the dual-tree complex wavelet transform is useful for finding the linear edges, the curvelet transform is useful for detecting the curve singularities, and the circlet transform is more appropriate for extracting the circular patterns in the images.

#### Transform Sub-Bands Formations Input Framework

**Figure 2:**
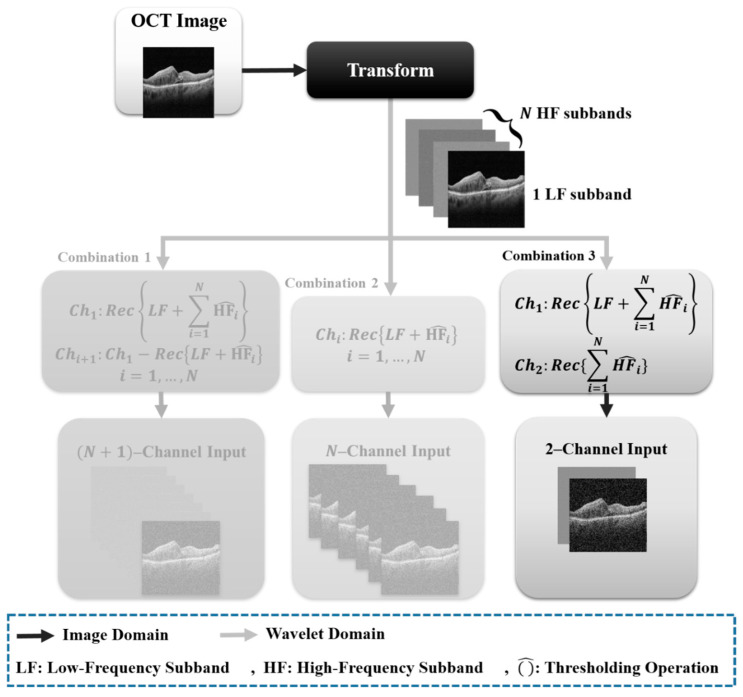
Different combinations of X-let subbands.

In prior work, we discovered that edge-based approaches out-performed context-based methods for cyst OCT segmentation when utilizing the DTCWT [24]. We opted to use the edge-based technique to compare the performance of various transformations. Figure 2 shows different edge-based combinations. Nevertheless, there are three edge-based approach options. In our previous work, we suggested that, of the three options for the edge-based technique, two of them, i.e., two channels and six channels in DTCWT, have better performance than the other options [24].

To compare different combinations in the edge-based strategy, we opted to apply a two-channel combination of different sub-bands as the input representation for OCT image segmentation because it provided a general form that did not cause hardware limitations. We tested different combinations of sub-bands to determine the optimal combination. Based on the results section, contourlet had better results; thus, we used two channels from this transform as the employed sub-bands.

**Figure 3:**
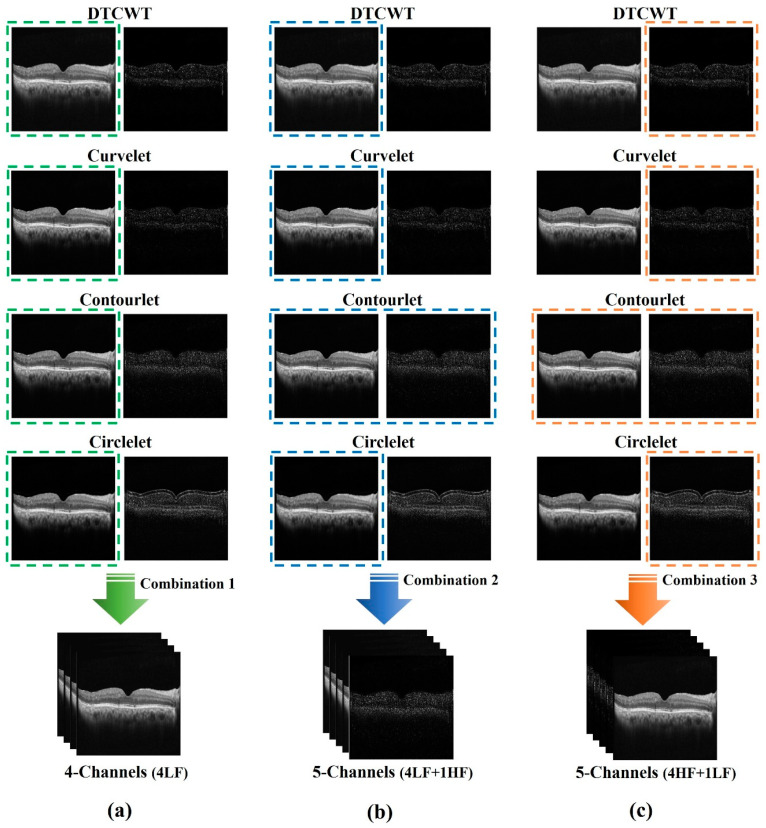
Proposed combinations that contain all two-channels of different sub-bands: (**a**) four-channel combinations, including four low-frequency channels of all transforms; (**b**) five-channel combinations, including four low-frequency channels of all transforms and high-frequency channels of contourlet; (**c**) five-channel combinations, including four high-frequency channels of all transforms and low-frequency channels of contourlet.

In Figure 3, different sub-bands of the two-channel of the transforms are shown. In the context of edge-based combination, the two-channel sub-bands referred to the decomposition of an input image into two sub-images. The first channel represented the low-frequency components of the image, while the second channel represented the high-frequency components that are extracted from band-pass and high-pass sub-bands. In the given context, the first channel image in the figure contained the low-pass filtered version of the original image, which represented the smoother regions. On the other hand, the second channel image contained the high-pass filtered version of the original image, which represented the edges and details. The high-pass filter enhanced the edges and details by highlighting the intensity variations in the image.

The contourlet transform provided a richer representation of edges in the image. It extracted edges at multiple scales and orientations and provided a sparse representation of the image. The two-channel combination of contourlet sub-bands enhanced the edge information and provided a better input representation for OCT image segmentation.

Combining different transformation sub-bands based on two-channels was useful for OCT cyst segmentation. In OCT images, different sub-bands or channels of a transform could capture different features of the cysts, such as their texture, shape, and size. By combining these sub-bands, we created a more comprehensive representation of the cysts, which could potentially improve the accuracy of segmentation. Based on the results sections, we suggested three different combinations for X-lets based on the two-channel combination. These combinations contained different transforms. The first combination, as shown in the first column of Figure 3, utilized the low-pass of both channels, as low-pass images produce better results than other sub-bands due to their similarity to the original image.

The second combination, as shown in the second column of Figure 3, used all five channels, which included four low-pass sub-bands and the edges obtained from the contourlet sub-bands. Finally, the third combination, as shown in the third column of Figure 3, employed all five channels, including the edges of all sub-bands and the low pass of the contourlet.

Many ways of denoising could be used in the reconstruction of OCT images. In this study, without any need to add any further denoising algorithm, a simple soft threshold-based denoising algorithm was utilized for all high-pass sub-bands to reduce noise in OCT images during the cyst segmentation process [46].

### 3.3. Network

As stated in the literature, the Trans-Unet model, which was built on transforms, out-performs other models in different image segmentation tasks [24]. The Trans-Unet model combined the strong properties of the transformer design, which is used extensively in natural language processing, with the advantages of the Unet architecture, which is frequently utilized in medical image segmentation applications. The transform design enables the model to more effectively capture global dependencies and long-term contextual information in the input data, while the Unet architecture offers a robust foundation for collecting local image attributes and spatial interactions. By merging these two architectures, the Trans-Unet model could attain state-of-the-art performance on various medical image segmentation tasks, making it a potential method for future study in this area.

**Figure 4:**
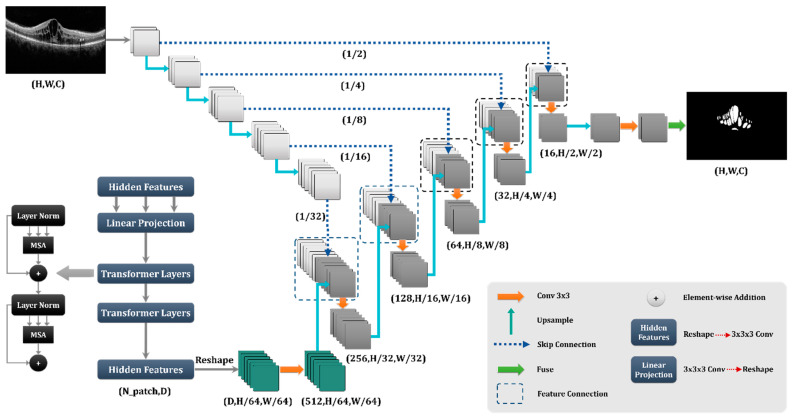
Proposed network for the X-let analysis.

The Trans-Unet with Figure 4 architecture was found to have the best performance in both qualitative and quantitative evaluation metrics that compared other proposed Unets and architectures. This finding meant that the Trans-Unet produced the most accurate segmentation results and showed better consistency in different datasets. Therefore, we implemented Trans-Unet as a base network.

### 3.4. Metrics

It is important to evaluate the performance of any segmentation method using both quantitative and qualitative metrics. Quantitative metrics provide numerical measures of performance, while qualitative metrics involve visual inspection of the segmented images for overall quality and consistency. Using quantitative and qualitative indicators, we gained a thorough transformation of the strengths and limitations of specific formations and made informed decisions about how to improve them to gain the best possible outcomes. In this section, we introduce these methods.

#### 3.4.1. Dice Score

The F1-score, or Dice index, is a performance indicator used to quantify the predictive accuracy of a model. It considers both a model’s accuracy and recall, and delivers a single scalar rating to represent overall performance. The harmonic mean of accuracy and recall is used to calculate the F1-score, with a higher score suggesting that the model makes fewer false positive or false negative predictions. The F1-score is a helpful statistic for unbalanced datasets, since it gives a more thorough assessment of the model’s performance than accuracy alone. It calculates scores by using the below equation:

(2) $$\mathrm{dice}=\frac{2|A⋂B|}{\left|A|+|B\right|}.$$

#### 3.4.2. Jaccard Index

The Jaccard index, also known as the Jaccard similarity or the Jaccard coefficient, is a statistic used to assess the similarity of two sets of data. It is determined by dividing the size of the sets’ intersection by the size of the sets’ union. The Jaccard index is a number between 0 and 1, with 1 representing total similarity and 0 representing no similarity between the sets. The Jaccard index is often used in image segmentation tasks to determine how successfully the anticipated segmentation mask coincides with the ground truth mask, and it calculates the score using the below equation:

(3) $$J\left(A,B\right)=\frac{\left|A\cap B\right|}{\left|A\cup B\right|}.$$

#### 3.4.3. Sen and FPR

The number of pixels in the image that are properly and mistakenly projected as tumor pixels is the TP, which is equivalent to Equation (4) (True Positive), and FP, which is (False Positive). N is the total number of non-tumor pixels, and P is the number of tumor pixels in the image. These metrics are applied to the qualitative result. Due to their greater sensitivity and lower FPR, the metric shows better performance.

(4) $$Sen\left(\%\right)=\frac{TP}{P}\times 100,FPR\left(\%\right)=\frac{FP}{N}\times 100.$$

### 3.5. Loss Function

In deep learning, a loss function is a mathematical function that computes the difference between the expected and actual target outputs. A deep learning model’s goal is to reduce the loss, which indicates the difference between anticipated and actual values. The loss function guides the optimization process, which adjusts model parameters to minimize loss. The loss function is chosen based on the kind of task, such as regression, classification, or segmentation. Deep learning often employs loss functions, such as mean squared error, cross-entropy, and the Dice similarity coefficient. In this paper, we propose the Tversky loss function because of its unbalanced dataset, and during the task, we find out new loss functions for the unbalanced datasets. 

#### Tversky Loss Function

Tversky Loss, also known as the Tversky index, is a popular loss function utilized in deep learning for semantic segmentation problems. This loss function aims to measure the similarity between two sets, the predicted segmentation, and the ground truth. The Tversky Loss function is unique in its calculation, as it employs a weighting factor that considers the false positive and false negative rate to balance the trade-off between precision and recall. The Tversky Index (TI) is an asymmetric similarity measure that combines the dice coefficient and the Jaccard index. This function is particularly useful when dealing with imbalanced datasets, where the ratio of positive to negative samples is uneven. The Tversky Loss function can be optimized using gradient descent algorithms, and is capable of providing highly accurate results in image segmentation tasks [47].

(5) $$I=\frac{TP}{TP+\alpha FN+\beta FP}.$$

## 4. Results

In this study, several deep learning architectures with different sub-band inputs and conditions were tested. These architectures were written in Python, and the deep learning models were trained and evaluated using a computer equipped with 64 GB of RAM, two parallel GEFORCE GTX 1080 Ti GPUs, and an i7 core 7th generation CPU. On PCs, Cuda version 10 and cuDNN version 7.5 were used. 

In our study of fluid localization in DME and AMD patients, various state-of-the-art techniques for fluid segmentation were implemented with Trans-Unet optimal parameters. In the first step, different transformations were applied for OCT semantic segmentation to determine the best-fit transform for the OCT semantic segmentation. To ensure fair comparisons, a fixed 150 epoch was set for the Trans-Unet selection; after that, suggested different combination transforms were tested with different transforms and the original image. In all networks, we tried some fixed parameters, as shown in Table 2:

### 4.1. Normal Condition

In this part, we propose to conduct a comparative analysis between different X-let combinations and simple image input. The purpose of this analysis may be to evaluate the effectiveness of different X-let combinations and determine the most suitable approach for the task. The comparison may involve using various evaluation metrics, such as F1-score, Jaccard, and loss curve, to measure the performance of each approach; the qualitative results are also evaluated. The comparisons were made between a simple image (the original OCT image), two channels of different sub-bands (i.e., using a combination of two sub-bands generated by DTCWT, curvelet, contourlet, or circlet transforms), and a combination of low-pass images of different transforms with high passes in various formations. This comparison is aimed at identifying the most effective representation for segmenting cysts in OCT images using their proposed deep learning model. The results of this analysis help us to make informed decisions when choosing the best sub-bands for the semantic segmentation task involving the proposed methods.

Table 3 demonstrates the performance of different X-let combinations based on Jaccard and Dice metrics. In this context, the results suggest that the DCWT-based combination outperforms the other combinations in the training phase, especially the original image. This result means that combining different transforms with the deep learning model improves the accuracy of semantic segmentation for OCT images. Moreover, the validation of the combination with 5-ch-hh is better than with other transforms. The best results are bolded in each column. 

**Figure 5:**
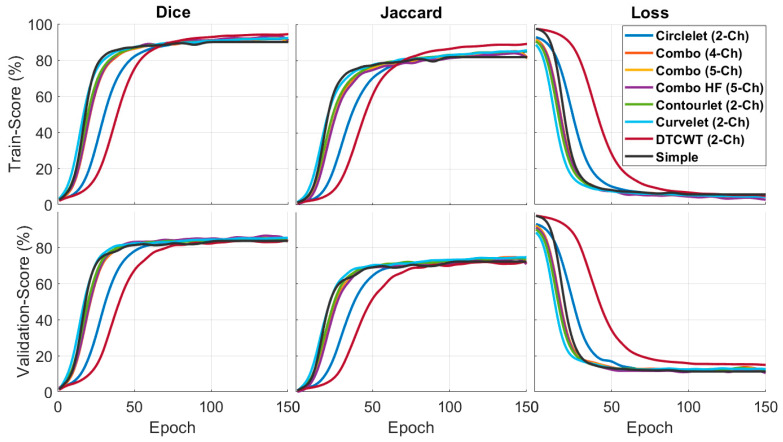
Quantitative outcomes of different X-lets sub-bands compared to simple image input.

Figure 5 compares the quantitative results of the X-let-based framework approach with other methods in terms of F1-score and Jaccard metrics. According to the statement, the DTCWT approach showed better performance than the other methods in the training phase, especially in comparing a simple image and the validation phase. Combination of the best sub-bands achieved the best performance in the suggested formations. This result means that the contourlet-based approach is more effective in segmenting the desired features, as indicated by the higher F1-score and Jaccard values.

**Figure 6:**
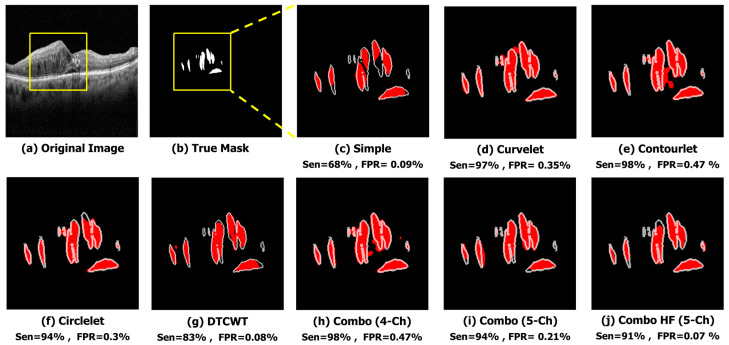
Qualitative result of different sub-bands using sample image. (**a**) Original Image. (**b**) True Mask. (**c**) Simple (Sen = 68%, FPR = 0.09%). (**d**) Curvelet (Sen = 97%, FPR = 0.35%). (**e**) Contourlet (Sen = 98%, FPR = 0.47%). (**f**) Circlelet (Sen = 94%, FPR = 0.3%). (**g**) DTCWT (Sen = 83%, FPR = 0.08%). (**h**) Combo (4-Ch) (Sen = 98%, FPR = 0.47%). (**i**) Combo (5-Ch) (Sen = 94%, FPR = 0.21%). (**j**) Combo HF (5-Ch) (Sen = 91%, FPR = 0.07%).

The qualitative results of a sample image in Figure 6 indicate that the X-let method performs better compared to other methods, such as applying Trans Unet on the simple image input. In this case, it suggests that all X-let transforms result in better performance compared to the simple image, with contourlet especially outperforming the others, and the five-channel combinations produce a more accurate and visually pleasing segmentation of the sample image. This fact can be useful for applications where high-quality visual results are important, such as medical imaging or computer vision.

**Figure 7:**
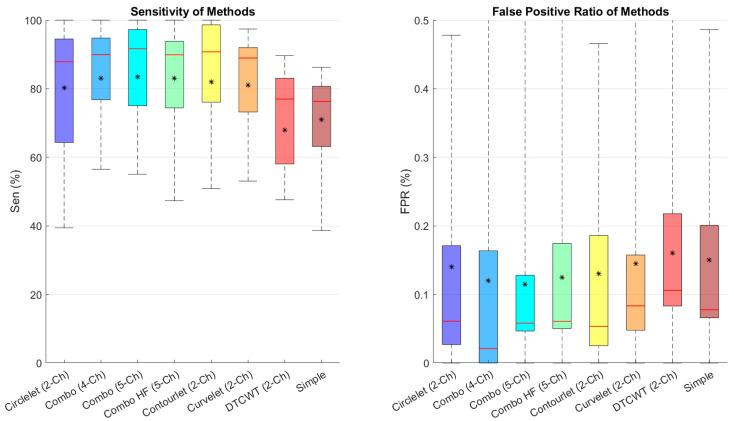
Boxplot of comparative examination of transformation segmentation of diverse transforms with different transformations. Star and redline show mean and median scores in each box, respectively.

Figure 7 depicts the sensitivity and FPR of different combinations, showing that the five-channel decomposition using high-pass sub-bands of contourlet transform performs better in finding cysts and that contourlet is the best transform between the suggested transforms.

### 4.2. Noisy Condition

In this section, we compare the performance of different combinations of transforms and their transformations on the segmentation of noisy OCT images. The proposed combinations were based on the contourlet, dual-tree complex wavelet, and curvelet transforms.

We evaluate the performance of the different combinations based on Dice and Jaccard metrics on the noisy dataset. Table 4 shows the results of two-channel combinations and combinations of various transforms. The two-channel contourlet has better performance between different transform sub-bands. The five–channel combination out-performs the other combinations in terms of Dice and Jaccard. This combination achieves a 79.2 on the Dice index across noisy conditions. The best results are bolded in each column. 

The second-best combination is related to the dual-tree complex wavelet transform-based sub-bands. 

**Figure 8:**
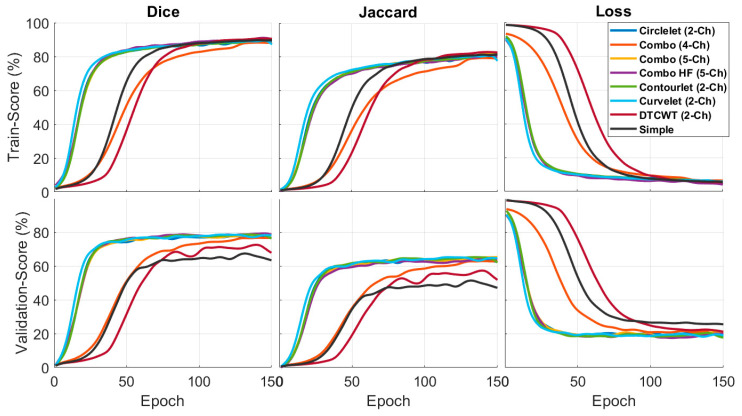
Quantitative results of the different X-lets sub-bands in noisy condition.

The performance of different sub-band combinations in noisy conditions is evaluated in Figure 8. The results are analyzed in terms of Dice and Jaccard, which shows that five-channel with high passes has better performance than the other transforms.

**Figure 9:**
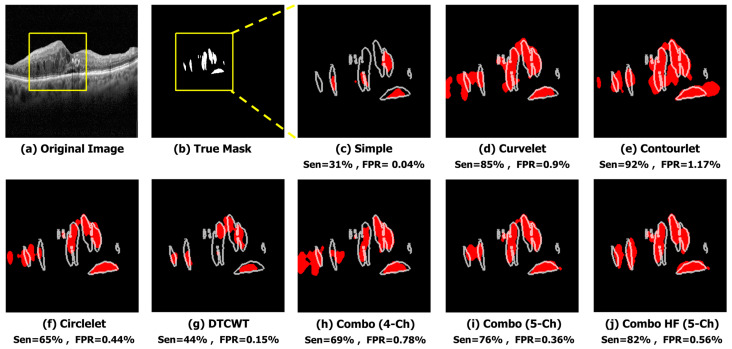
Qualitative result of different sub-bands in the noisy condition. (**a**) Original Image. (**b**) True Mask. (**c**) Simple (Sen = 31%, FPR = 0.04%). (**d**) Curvelet (Sen = 85%, FPR = 0.9%). (**e**) Contourlet (Sen = 92%, FPR = 1.17%). (**f**) Circlelet (Sen = 65%, FPR = 0.44%). (**g**) DTCWT (Sen = 44%, FPR = 0.15%). (**h**) Combo (4-Ch) (Sen = 69%, FPR = 0.78%). (**i**) Combo (5-Ch) (Sen = 76%, FPR = 0.36%). (**j**) Combo HF (5-Ch) (Sen = 82%, FPR = 0.56%).

For qualitative evaluation, the segmented OCT images are visually inspected and compared to the ground truth images shown in Figure 9. We found that the combination of low-pass contourlet and high-pass sub-bands provided the best results in terms of visual quality.

**Figure 10:**
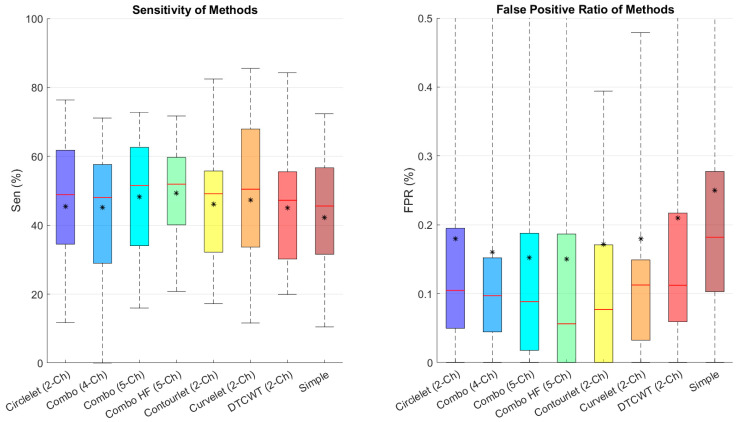
Boxplot of comparative examinations of transformation segmentation of different transforms with different transformations in noisy condition. Star and redline show mean and median scores in each box, respectively.

In order to analyze the FPR and sensitivity in the noisy condition, we calculated and compared these metrics in Figure 10. We found that the combination of low-pass contourlet and all sub-bands’ edges had the highest results.

## 5. Conclusions and Future Work

OCT is an imaging technique that produces high-resolution cross-sectional images of biological tissues. Accurate and efficient segmentation of cystic spaces in OCT images can aid clinicians in diagnosing and monitoring retinal diseases and can help them to make correct treatment decisions. This paper focused on finding the best sparse transform between the suggested transform for automated OCT cyst segmentation and using their combination to find the optimal formation for the OCT cyst semantic segmentation. Deep learning-based OCT cyst segmentation can also potentially reduce the workload of ophthalmologists and improve patient care. To achieve this goal, Trans-Unet is used as the base network. 

The proposed deep learning method for OCT cyst segmentation has significant clinical potential. Accurate and efficient cyst segmentation can aid ophthalmologists in the diagnosis and monitoring of a variety of eye diseases, including macular edema and age-related macular degeneration, which are major causes of blindness and visual impairment. Early detection and precise monitoring of cysts are crucial for preventing vision loss in affected patients. Automated segmentation using deep learning models reduces the need for manual segmentation, which is a time-consuming and tedious process. It can also assist ophthalmologists in making more informed decisions about patient care. As demonstrated in this paper, the high accuracy and efficiency of deep learning models in segmentation tasks make them a promising tool for improving the diagnosis and treatment of eye diseases. Therefore, the proposed method has significant clinical potential and can improve patient outcomes in the field of ophthalmology. For example, this method could be an efficient way to measure fluid differences in patients undergoing anti-VEGF therapy. By automatically segmenting the areas of fluid accumulation in the retina, the method can provide a quantitative measure of the changes in fluid volume over time. This approach could be particularly useful for monitoring the effectiveness of anti-VEGF therapy, which aims to reduce fluid accumulation in the retina.

The proposed network was incredibly successful in OCT semantic segmentation; however, it faces several challenges in terms of the noisy and extracting edges. Deep learning methods, such as the X-let sub-band combinations based on Unet proposed in this paper, can significantly improve the accuracy and efficiency of the segmentation process. These sub-bands can be used as inputs to adapt the deep learning model for semantic segmentation, which can improve the model’s performance by providing more comprehensive information about the image. Different X-lets refer to different transforms that can be applied to images for feature extraction. The different X-lets that were suggested in this paper included the curvelet transform (this transform is used to extract curved features from an image), contourlet transform (this transform is used to extract features from an image that has edges and contours), circlet transform (this transform is used to extract features from an image that has circular symmetry), and dual-tree complex wavelet transform (this transform is used to extract features from an image at multiple scales and orientations). By combining the features extracted from multiple transforms, it is possible to improve the accuracy of semantic segmentation in OCT images. Using the combination of X-lets involved in the proposed deep learning method, this paper illustrated the model’s ability to segment fluid regions in OCT images, especially in noisy conditions. Therefore, the use of X-lets in OCT cyst segmentation can lead to more accurate and reliable diagnosis and treatment of retinal diseases.

Future work can include fine-tuning the parameters, incorporating regularization terms in both the loss function and a hyperparameter model, using other X-lets, selecting the optimal combination among X-lets, and introducing a unique loss function for cyst segmentation. Additionally, a novel Unet architecture based on sub-bands can be proposed, and different X-let sub-bands can be investigated using other novel networks.

The mixture of experts is another promising suggestion for improving the performance of the OCT semantic segmentation task. This approach involves training multiple specialized networks, or experts, to perform effectively on different subsets of the data. The outputs of these experts are then combined to yield more accurate predictions than any of them could yield individually. By leveraging the strengths of different networks for different parts of the image, the mixture of experts can potentially improve the segmentation performance, especially in challenging cases where a single network may struggle.

## Figures and Tables

**Table 1:** Comparison of different sparse-based deep learning architectures discussed in related work.

Framework	X-Let Transform	Type	Input	Key Features	Reference
DTCWT-CNN	Dual-tree complex wavelet transform (DTCWT)	Network structure	Image	DTCWT used in CNN layers instead of the max-pooling layer for improved segmentation accuracy	Hongya Lu et al. [35]
Wavenet Unet	Wavelet transform	Network structure	Image	Modified Unet model with Wavenet wavelet layer for improved segmentation	Qiufu Li et al. [39]
Max-Wavelet Pooling	Wavelet transform	Network structure	Image	Multi-pooling approach combining wavelet and classic pooling for improved performance	Brito et al. [40]
Wavelet Unet	Wavelet transform	Network structure	Image	Wavelet transform used in Attention Unet for concrete crack segmentation	Guiyi Yang et al. [36]
Contourlet Attention Unet	Contourlet transform	Input framework	Sub-bands	Contourlet sub-bands are used as inputs in marine raft segmentation networks based on Attention Unet	Zhang et al. [37]
3D Discrete Wavelet Transform	3D Discrete Wavelet Transform	Input framework	Sub-bands	3D Discrete Wavelet Transform used as the input image for polarimetric SAR images	Bi, H. et al. [38]
Attention Unet with DTCWT	DTCWT	Input framework	Sub-bands	Applied different methods for OCT cyst segmentation	Darooei et al. [24]
Proposed Optimal X-let Transform	Combination of different sub-bands transforms (DTCWT, contourlet, curvelet, circlet)	Input framework	Sub-bands	Novel optimal X-let transform for improved segmentation performance	This paper

**Table 2:** Trans-Unet networks fixed parameters.

Loss	Optimizer	Number of Filters	Epochs
Tversky (*α* = 0.8, *β* = 0.2)	Adam (learning rate = 1 × 10^−4^)	(8, 16, 32, 64, 128)	150

**Table 3:** Comparison of different X-lets combinations with Trans-Unet using simple image.

Transform	Dice (Validation)	Jaccard (Validation)
Simple Image	90.73 (82.59)	82.08 (72.78)
Curvelet (2-ch)	92.32 (85.64)	85.84 (75.29)
Contourlet (2-ch)	92.07 (85.25)	85.41 (74.73)
Circlet (2-ch)	92.06 (85.54)	85.4 (75.07)
DTCWT (2-ch)	**94.52** (84.23)	**89.64** (73.67)
Combination 1 (4-ch)	92.09 (85.80)	85.45 (75.6)
Combination 2 (5-ch)	93.12 (86.21)	84.21 (73.82)
Combination 3 (5-ch with-hh)	93.4 (**86.5**)	84.0 (**73.9**)

**Table 4:** Comparison of different X-lets combinations with Trans-Unet in noisy condition.

Transform	Dice (Validation)	Jaccard (Validation)
Simple Image	89.8 (67.4)	81.6 (52.4)
Curvelet (2-ch)	89.4 (78.5)	81 (65.4)
Contourlet (2-ch)	89.7 (78.6)	81.5 (65.4)
Circlet (2-ch)	88.9 (77.6)	79.4 (63.1)
DTCWT (2-ch)	90.6 (72.5)	**83** (57.4)
Combination 1 (4-ch)	88.3 (76.7)	79.4 (63.1)
Combination 2 (5-ch)	90.2 (78.4)	82.3 (65.5)
Combination 3(5-ch with-hh)	**91.1** (79.2)	**81.6** (65.8)

## Data Availability

Not applicable.
